# The Kiss-and-Run Model of Intra-Golgi Transport

**DOI:** 10.3390/ijms13066800

**Published:** 2012-06-05

**Authors:** Alexander A. Mironov, Galina V. Beznoussenko

**Affiliations:** IFOM Foundation, FIRC Institute of Molecular Oncology (IFOM-IEO Campus), Via Adamello 16, 20139, Milan, Italy

**Keywords:** intra-Golgi transport, Golgi apparatus, vesicular model, cisterna maturation, kiss-and-run model

## Abstract

The Golgi apparatus (GA) is the main station along the secretory pathway. Mechanisms of intra-Golgi transport remain unresolved. Three models compete with each other for the right to be defined as the paradigm. The vesicular model cannot explain the following: (1) lipid droplets and aggregates of procollagen that are larger than coatomer I (COPI)-dependent vesicles are transported across the GA; and (2) most anterograde cargoes are depleted in COPI vesicles. The compartment progression/maturation model has the following problems: (1) most Golgi-resident proteins are depleted in COPI vesicles; (2) there are no COPI vesicles for the recycling of the resident proteins in the *trans*-most-Golgi cisterna; and (3) different proteins have different rates of intra-Golgi transport. The diffusion model based on permanent inter-cisternal connections cannot explain the existence of lipid, ionic and protein gradients across the Golgi stacks. In contrast, the kiss-and-run model has the potential to explain most of the experimental observations. The kiss-and-run model can be symmetric when fusion and then fission occurs in the same place, and asymmetric when fusion takes place in one location, whereas fission takes place in another. The asymmetric kiss-and-run model resembles the carrier maturation mechanism, and it can be used to explain the transport of large cargo aggregates.

## 1. Introduction

The mechanisms of intracellular trafficking remain under discussion within the scientific community. Currently, there are three main models of intra-Golgi transport under consideration: (1) the vesicular model; namely, transport by anterograde, COPI-dependent vesicles; (2) the compartment progression/maturation (CPM) model, where recycling of resident Golgi proteins occurs through retrograde vesicles or inter-compartmental connections; and (3) the diffusion model. The general functional principles of these models have been described in several previous reports [1–4].

The demonstration that most cargoes are depleted in COPI vesicles (for example, a 3-fold depletion of biosynthetic transmembrane proteins (type I/II/III) from entering budding COPI vesicles is observed [5,6]; reviewed in details in [3]) and that cargo aggregates that are incompatible with the size of COPI vesicles are transported through the Golgi apparatus (GA) [7] has allowed the rejection of the vesicular model. Although Orci *et al*. [8] reported enrichment of proinsulin in COPI vesicles, their data contain information that can be interpreted in the opposite manner. For instance, “Table 3” in Orci *et al*. [8] shows the labeling densities of proinsulin in COPI-coated buds at the *cis* pole of the GA as 166 ± 37, and at the *trans* pole as 145 ± 58. In the cisternae of the *cis* half of the GA the density is 261 ± 37, and for the *trans* half, 235 ± 32. To fit these data to the vesicular model, Orci *et al*. [8] proposed two populations of vesicles, and only by using this assumption could they estimate that there is 1.4–1.8-fold enrichment of proinsulin in COPI-dependent anterograde vesicles.

Mathematical modeling has revealed that good performance of the CPM model with vesicle-mediated recycling is possible only when the Golgi resident proteins (GRPs) are concentrated in retrograde vesicles [9]. Indeed, Martinez-Menarguez *et al*. [10] demonstrated that *in situ*, mannosidase II (ManII) is slightly (1.16-fold) more concentrated in COPI-coated peri-Golgiolar round profiles. However, other studies have reported a depletion of ManII in peri-Golgi round profiles [11–14].

COPI vesicles are depleted of several GRPs that should undergo recycling; namely, TGN38, ERGIC53, p24 and syntaxin 5 [6]. This means that after several rounds of transport and recycling, these proteins would be shifted to the plasma membrane or endosomes. On the other hand, if one Soluble NSF Attachment Protein Receptor (SNARE) from the same SNARE complex is relatively more concentrated in COPI vesicles, very soon the stoichiometric ratio of the SNAREs within the same SNARE complex would be impaired at the level of the GA. However, this is not the case. Recently, we have seen that nucleotide sugar transporters are strongly depleted in COPI vesicles [15]. Altogether these data argued against the CPM model.

There is additional evidence against the role of COPI vesicles as retrograde carriers within the GA:

1. Some GRPs, such as sialyl transferases [16,17] and fucosyl transferases [18] are present in the *trans*-most cisterna of the GA. However, there are no COPI-coated buds on the *trans*-most cistern [19,20]. Therefore, it is not clear how these GRPs will undergo recycling from the *trans*most cisterna.
2. One of the problems of the CPM model is the mechanism of delivery of COPI vesicles filled with GRPs to the proximal compartment for the recycling of GRPs in yeast. In mammalian cells, COPI vesicles can be found as “strings” [21]. In contrast, in the yeast *Saccharomyces cerevisiae*, the different Golgi compartments are localized separately from each other, and hence they are divided by significant space. How COPI can be directed to the correct compartment in this case is not clear. Thus, it is necessary for a specific mechanism for the directed movement of COPI vesicles in yeast, because if their diffusion was not limited and targeting systems were lacking, COPI vesicles would fill the entire cytosol. However, this is not the case. Moreover, it has been shown that in mammalian cells, the ability of >50-nm particles (let us say, COPI vesicles) to diffuse throughout the cytosol is very limited [22].
3. The movement of ManII during the polarization of Golgi stacks after washout of brefeldin A occurs when the Arf1/COPI machinery is blocked [23], which argues against the role of COPI vesicles for recycling of GRPs.
4. In yeast containing the temperature-sensitive αCOP mutant ret1-1, which is known to be defective in COPI vesicle formation at the restrictive temperature, 37 °C [24], maturation of the Golgi compartments (as the replacement of the color particular to the proximal compartment by the color characteristic of the distal Golgi compartment) still occurs, even at the restrictive temperature [25].
5. Finally, the concentration of regulated secretory proteins within the Golgi stacks [26] cannot be explained within the framework of the CPM model [27].

Thus, there is a lot of experimental evidence against the classical variant of the CPM model as the main mechanism of intra-Golgi transport.

However, it is possible to imagine that the recycling of GRPs might occur not only by COPI-dependent vesicles, but also through intercisternal connections [28,29]. This proposal gives the second variant of the CPM model. This model is Golgi-matrix dependent, as it should be a mechanism for the recycling of the matrix receptors, or the GRPs *per se* would be involved in the attachment of cisternae to each other. Also, this mechanism should provide a higher affinity for the matrix receptor at the *cis* side of a Golgi stack, for instance, for the recycling of the matrix proteins from the *trans* to the *cis* side of the stack. Alternatively, the cytosolic tails of *cis*-GRPs should have a higher affinity for each other than for the cytosolic tails of the *trans* enzymes. Thus, if it is possible to acutely inhibit the function of the Golgi matrix, the transport of cargo through the GA should be blocked. However, this mechanistic explanation faces some difficulties. GM-130, GRASP-65 and GRASP-55 are not found inside the Golgi stacks between all of the medial cisternae [30]. If a mechanism that forces the *cis* membrane to move towards the *cis* side existed, the model should be sensitive to the absence of stacks. However, in insect S2 cells, a double depletion of dGRASP and dGM130 led to the quantitative conversion of the Golgi stacks into clusters of vesicles and tubules, often featuring single cisternae. However, this disruption of the Golgi architecture was not accompanied by the disorganization of endoplasmic reticulum (ER) exit sites, or the inhibition of anterograde transport [31].

Thus, normal stacks are not necessary for intra-Golgi transport. Moreover, this model cannot explain fast intra-Golgi transport in *S. cerevisiae*, where there are no Golgi stacks as such. Next, this model requires the departure of post-Golgi carriers from the *trans*-most cisterna. However, this departure occurs from the last three *trans* cisternae [20]. Another problem with this model is that intercisternal connections are very narrow and curved [28,32], and they are not always present within the Golgi stacks [33]. Recently, the existence of ultra-thin intercisternal connections was confirmed using vitreous sections from samples that had never been subjected to any kind of chemical treatment [34]. The diffusion of membrane proteins through these intercisternal connections might be quite different from that of luminal cargoes and ions, due to problems of the geometry of membrane proteins, and especially their aggregates, which might not be able to diffuse along highly curved tubes.

Recently, the diffusion model has undergone a revival, based on kinetic fluorescence analysis of cargo exit from the Golgi zone [35]. There is evidence in favor of the diffusion model. Pagano *et al*. [36] demonstrated that externally-added lipids can move from the plasma membrane to the GA and then to the ER even in aldehyde-fixed cells, and that OsO_4_, which freezes the lateral diffusion of lipids, blocks this movement. This lipid movement in aldehyde-fixed cells suggests that there are physical continuities between the GA and the plasma membrane. Furthermore, dicumarol, an inhibitor of brefeldin-A-induced ADP-ribosylation, specifically destabilizes Golgi tubules and delays intra-Golgi traffic [37]. In contrast, after activation of protein kinase A, the GA becomes interconnected and intra-Golgi transport is accelerated [38]. Some lipids (e.g., phosphatidylethanolamine, diacylglycerol) can be easily transported along the exocytic pathway at low temperatures when traffic (including the formation of vesicles) and endocytosis are inhibited [39,40]. Finally, tubules also contain secretory proteins [29,41]. These reports have thus indirectly suggested that intercisternal connections are important for intra-Golgi transport.

It has been established that cargo exit from the area of the GA appears to follow exponential kinetics, as reported by Patterson *et al*. [35]. Under these circumstances, and according to kinetic principles, each molecule of a diffusible cargo should have almost unlimited access to any Golgi compartment. If every such molecule in each cisterna has the same (or almost the same) probability to exit from the GA, export from the GA can be considered as a first-order process, which would follow an exponential curve [5,35]. Therefore, the diffusion mechanism was proposed as the main mechanism of intra-Golgi transport [35].

One of the prerequisites of the diffusion model is the necessity for stable intercisternal connections. Intercisternal connections have been rarely observed by conventional electron microscopy, and even in three-dimensional studies of regulated secretory cells [42,43], which suggests that these connections are normally short-lived. Intercisternal connections have been seen between the ER and the *cis*-Golgi at steady-state [44] and after a 15 °C temperature block [28], and between the *trans*-most cisterna and the *trans*-Golgi network (TGN) [45]. Branching of cisternae has been shown by Clermont *et al*. [46]. In the GA of early spermatids, such connecting membranous tubules not only bridge saccules of adjacent stacks, but also bridge saccules of the same stack [46]. The interlacing of cisternae was demonstrated by Griffiths *et al*. [47]. Sometimes, Golgi cisterna can surround two other parallel cisternae in a horseshoe shape [48].

The three-dimensional structure of the GA in many cell types shows a spiral-like organization [46,49,50]. In transmission electron microscopy micrographs, the cisternae also appear to be separate; however, using scanning electron microscopy, it is possible to see continuities, and even the formation of a spiral structure from a single cisterna, which in section looks like a typical stack [50]. Thus, transport would occur along a tangential direction to the GA.

Intercisternal connections located at different levels of the GA have been observed at steady-state [34]. Intercisternal connections are also more abundant in transporting Golgi stacks [28], and after stimulation of cell signaling [32]. When quick freezing and subsequent electron cryo-microscopy was applied, the intercisternal connections seen were very narrow [34].

One of the main problems of the lateral diffusion model is the existence of stable protein and lipid gradients along the membranes of the GA, and ionic gradients along the lumen of the GA [51,52]. The high rates of diffusion of lipids, ions and proteins argue against the diffusion model. The presence of SNARE complexes within all stages of the secretory pathway represents another caveat for the diffusion model.

Finally, there is the carrier maturation-progression model [2,3,53], which implies that cargo domains preserve their identity, thus remaining distinct and not intermixing with the Golgi domains containing Golgi resident proteins, such as Golgi glycosylation enzymes and nucleotide sugar transporters. However, megavesicles [54] are not used for intra-Golgi transport [53]. Instead, the fusion of a cargo domain with the distal cisterna occurs before its fission from the proximal one. In this case, there is no necessity for retrograde dissociation carriers (*i.e.*, vesicles or intercisternal connections). Importantly, this model does not need the recycling of the Golgi resident proteins from one cisterna to another. Observations based on 4Pi-microscopy and correlative light-electron microscopy have suggested that during synchronous intra-Golgi transport of the G-protein of VSVG, complete intermixing of the two domains containing VSVG and the Golgi resident proteins does not occur [55], which is in agreement with this model. However, the different rates of the passage of albumin and VSVG across the Golgi stacks argue against this model [29]. Similarly, this model cannot explain the role of intercisternal connections for intra-Golgi transport.

## 2. The Kiss-and-Run Model of Intra-Golgi Transport

However, all of the above-mentioned problems can be resolved within the framework of the kiss-and-run (KAR) model. Initially, the KAR model was proposed for the explanation of the phenomena related to the function of the synaptic vesicles, by Cecarelli *et al*. [56]. Then, the KAR model was implied for fusion between endosomes and lysosomes [57], secretory granules and the plasma membrane in neuroendocrine cells (reviewed in [58]) and post-Golgi carriers and the plasma membrane [59]. Finally, we proposed that the KAR model could explain intra-Golgi transport [3].

**Figure 1:**
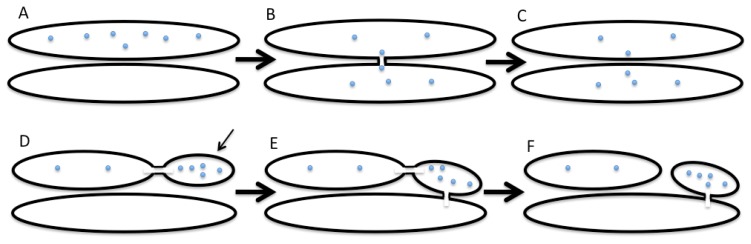
Two variants of the kiss-and-run model. Upper panel: the symmetric kiss-and-run model. Lower panel: the asymmetric kiss-and-run model. (**A**) Molecules of a cargo (blue dots) are present in the upper cisterna; (**B**) Fusion between two cisternae and diffusion of cargo to the lower cisterna; (**C**) Fission of the connection between two cisternae. Now, part of cargo molecules is in the lower cisterna; (**D**) Molecules of a cargo are partially concentrated in a domain (thin arrow) of the upper cisterna. The domain is connected with the cisternae by thin tubule; (**E**) Fusion between the right domain and the lower cisterna; (**F**) Fission of the tubule connecting the upper cisterna and the domain. Now, the domain belongs to the lower cisterna being connected with it and separated from the upper cisterna.

The KAR model might exist in two forms. The first variant of the KAR model assumes that two compartments fuse with each other, and then the neck connecting these two compartments undergoes fission. During some of this period, the lumens of the two juxtaposed compartments will be connected and will then undergo disconnection. During this period solutes can diffuse from the lumen of one compartment to the lumen of the other compartment (Figure 1).

The asymmetric KAR model is similar to the carrier maturation-progression model, which easily explains all of the data related to the intra-Golgi transport of large cargo aggregates [3]. The asymmetric KAR model assumes that after the fusion, the following fission might occur at another site that allows a part of one membrane to cross from one compartment to another. In other words, the temporal fusion between two organelles at one site with the following fission at another site allows for some membrane displacement from one organelle to the other. The asymmetric KAR model is similar to the carrier-maturation model (see above), which could be considered as a limited case of the KAR model. For instance, procollagen-I-containing distensions localized within the *cis*-most cisterna or the first medial cisterna can fuse with the rim of the last medial cisterna, and then be separated from the original cisterna along the row of pores that separate the distension from the rest of the cisternae [20].

The function of the KAR model is similar to the function of the lymphatic duct, which can raise lymph up to the pressure of 1.5 m in height, even though the capacity of each segment to contract is very weak. The contraction wave moves from the upper part of the lymphatic duct towards the lower part. When the single layer of smooth muscle cells lining the upper segment of the lymphatic duct contracts, the valve below the segment becomes closed and the lymph goes up. Then, contraction of the second segment induces the closure of the underlying valve, and the lymph goes to the previously emptied and most upstream segment of the lymphatic duct, and so on. The fission mechanism has the role as that of the underlying valve.

For its normal function, the KAR model has several requirements:

1. It requires a mechanism to ensure vectorial transport of the membrane within the framework of the asymmetric KAR model or the carrier-maturation model;
2. There should be a mechanism for the concentration of the SNAREs in sites through which two compartments fuse with each other;
3. The cells have to have a mechanism to break these membrane connections;
4. To concentrate proteins in the distal Golgi compartments, there needs to be a mechanism that regulates the vectorial diffusion of proteins along intercisternal connections. For instance, this could be a gradient of ionic pumps or other protein machineries that can regulate the concentration of ions across the GA in order to create these gradients.

On the other hand, these tubules or connections should be transient and narrow, because if these membranous intercisternal tubules are narrow enough they will prevent retrograde diffusion of proteins that tend to aggregate under certain ionic conditions. Finally, the KAR model actually means that there is no necessity for specific retrograde transport, as this transport occurs simultaneously with anterograde transport.

## 3. Concentration of Soluble Cargoes and Golgi Glycosylation Enzymes

Is it possible to reach a significant level of cargo concentration within the framework of the KAR model? The answer, we argue, is yes. The algorithm, which could solve the problem, is the following: in any combination of the paired compartments, each distal compartment induced concentration of a protein within a pair. Let us assume that we have several compartments localized consecutively and very close to each other. Initially, there are connections between the first and the second, between the third and the fourth, and between the fifth and the sixth compartments. Due to their different abilities to pump protons, the acidity in each distal compartment will be higher than in any proximally-situated compartment. Thus, in the second compartment, the acidity is higher than in the first, in the fourth compartment, it is higher than in the third, and so on. For example, protons might have a role in the regulation of cargo oligomerization. In its monomeric state, a cargo might pass through narrow connections, whereas in its oligomeric state, it cannot. Simple calculations (see Table 1) show that in several rounds of fusion/fission events, the maximal concentration of a soluble cargo will be in the last compartment, and it can reach levels 7-fold greater and more. As such, one of the requirements would be a gradient of proton pumps, because if the source of protons is only in the most distal cisterna, the system will not work due to the greater rate of proton diffusion than protein diffusion. Within the framework of our model, the wave of concentration moves along the proximal-to-distal direction, whereas the resident proteins will move in the opposite direction.

The KAR model can explain the mechanisms of concentration of the regulatory secretion proteins. For such a system to work, the compartments of the distinct ionic environments must be narrowly connected to limit admixing, and the condensation of different regulatory secretion proteins along these interconnected, but distinct, compartmental domains must create a diffusion gradient along the tubular connections. On the other hand, if these intercisternal connections were permanent, it would be difficult to explain the increasing concentrations of the regulatory secretion proteins that were described by Oprins *et al*. [26].

The KAR model can also explain the concentration of the GRPs in the defined Golgi compartment if at the end of the GA, the ionic gradient is opposite. For instance, within the consecutive pairs, namely, ER–intermediate compartment, intermediate compartment–*cis*-most cisternae, *cis*-most cisterna–first medial cisterna, first medial cisterna–second medial cisterna, second medial cisterna–third medial cisternae, the ionic gradients are oriented towards the ER. The capacity of the next compartment to pump ions is thus higher than the capacity of the previous one. However, in the last two consecutive pairs next considered, namely third medial cisterna–fourth medial cisterna and fourth medial cisterna–*trans*-most cisterna, and in the more distal parts, the capacity of these distal compartments to pump protons is lower. Simple calculations following the transport rates show that if the GRPs depend on the capacity to pump protons, then their maximal concentration will very soon be in the third medial cisterna (see Table 2).

The KAR model can also explain the data of Matsuura-Tokita *et al*. [25] and Losev *et al*. [60] about the directionally of “maturation” of Golgi compartments. Indeed, Golgi membranes are filled with membrane proteins, and there is no empty space [61]. Thus, when two consecutive compartments fuse with each other, a membrane cargo protein from the proximal compartment should move to the distal compartment. Due to the absence of empty space there, this should be a process that includes the displacement of membrane-resident proteins situated within the distal compartment by the cargo protein. As a result, the resident protein that is situated in the distal compartment can be forced to move into the proximal compartment, and so the proximal compartment would acquire features of the two compartments. The membranes of the proximal, and not the distal, compartment then become composed of markers of the proximal (*i.e.*, red) and distal (*i.e.*, green) compartments, thus becoming yellow due to movement of the “green” protein from the distal compartment. Of interest is that a careful analysis of the movies presented by Matsuura-Tokita *et al*. [25] demonstrated that when the proximal compartment acquires the marker of the distal compartment, these two compartments are close to each other (during compartment collision, no space between these Golgi compartments is visible; these collisions are detected in 5 movies among 6 presented). Three-dimensional reconstruction has revealed that different Golgi compartments in *S. cerevisiae* are often connected [62], in agreement with the KAR model.

The directed movement of cargo-containing membrane domains across the GA can be explained by the following. If we assume that the fusion between the cargo domain and the Golgi cisterna is a stochastic mechanism, one mechanism would be the gradient of lipids, and a second would be the gradient of Ca^2+^ from the *trans* ER during intra-Golgi transport [63]. If the concentration of Ca^2+^ increases along the *cis*-to-*trans* direction, the probability for the cargo domain to fuse with the more distal cisterna would be higher. After its arrival at the *trans* side of the GA, the cargo domain would be captured by the *trans*-most cisterna, which is attached to the medial Golgi stacks by a Golgin-97-dependent attachment mechanism [64].

## 4. The Kiss-and-Run Model and the Fusion and Fission Machineries

One of the functions of COPI vesicles is the extraction of membrin (GOS27) and GOS28 from the cisternae to prevent their fusion and to inhibit intra-Golgi transport. In resting Golgi stacks, the number of COPI vesicles increases [15,28,64]. When the cargo arrives at the GA, the vesicles fuse with the Golgi cisternae because this arrival of cargo induces the release of Ca^2+^ from the Golgi membranes [63]. This fusion delivers Membrin and GOS28 back to them. Now, all of the SNAREs are present on the GA. As such, the COPI vesicles would inhibit intra-Golgi transport, by extracting Qb SNAREs from the Golgi membranes [3].

In the framework of the KAR model, the fission of membrane tubules that connect the different Golgi compartments becomes one of the most important mechanisms. One of the elements of the fission machinery might be COPI. Incubation of *in*-*vitro*-reconstituted Golgi tubules in the presence of GTPγS and cytosol leads to the transformation of tubules into bead-like structures, and then vesiculation [65].

The second element of this fission machinery might be COPI/brefeldin A ribosylation substrate (BARS or CtBP1). Recently, it has been shown that several proteins are apparently involved in such a fission mechanism. For BARS, the most convincing data relate to its role in the fission of COPI-coated buds from Golgi membranes. A BARS-dependent mechanism is also interchangeable with an endophilin A mechanism [66,67]. The same mechanism might be responsible for the fission of intercisternal connections. A further mechanism might be via dynamin, which is considered to be involved to the fission of clathrin-dependent vesicles from the plasma membrane. Finally, local temperature fluctuations might also regulate fission. COPI might serve (together with BARS) as a machinery that breaks intercisternal connections.

## 5. Potential Problems with the Kiss-and-Run Model

Thus, the KAR model can explain most of the present contradictions relating to intra-Golgi transport. However, several unclear questions remain to be answered:

1. Why does the exit of procollagen-I-GFP from the Golgi zone at steady-state occur according to an exponential decay, although the aggregates of procollagen-I-GFP cannot diffuse along the Golgi ribbon [35]? The reason for this might be the following. Most of the distensions containing procollagen-I are present within the TGN or the putative post-Golgi compartment and their emptying depends on the growth of microtubules. Indeed, as seen by figures 7, 8, 24 and 34 in Weinstock and Leblond [68], *in situ* at a steady-state, most of the procollagen-I distensions that are visible within the Golgi area are not integrated into the cisternae, but are localized out of the stacks, apparently within the putative post-Golgi compartment or the TGN.
2. How can significant penetration of VSVG be explained when a large amount of cargo moves across the GA? Indeed, several studies [35,69,70] have found significant penetration of cargo into the GA (a cargo quickly reaches the *trans*-side of the GA) when large amounts of cargo are accumulated just before the GA. For instance, according to Bergman *et al*. [71], VSVG passes through the GA in 1–3 min. In only 10 min after release from a 40 °C temperature block, endo-H-sensitive VSVG (not processed by the medial Golgi enzyme ManII) appears in clathrin-coated vesicles [72]. If we consider that clathrin-coated buds are found exclusively on the *trans* cisternae [19] and that VSVG undergoes folding over 4–5 min [73], this suggests that VSVG reaches the *trans*-most cisterna 6 min after the moment when the first VSVG molecules reach the *cis* Golgi. This is faster than the well-established time necessary to cross the GA (10–20 min [7,53]). On the other hand, it is known that all of the medial cisternae have a more or less similar SNARE composition [74]. If the amounts of cargo traversing the GA simultaneously are high, the SNAREs within the first cisterna will be consumed quickly, and thus these cargoes will be forced to fuse with the subsequent cisterna. Even when the cargo domain arrives at the *cis* side of the Golgi stack, it might fuse not only with the first, but also with subsequent cisternae, by mistake. Thus, the deep penetration of VSVG and procollagen I through the GA can be explained without the assumption that they diffuse across the Golgi stack.
3. Why does the diffusion of VSVG-GFP along the Golgi ribbon depend on the amount of VSVG-GFP passing through the GA [53]? Small domains of VSVG might not be able to diffuse through the Golgi enzymatic domains that are densely filled with the molecules of the GRPs [53]. However, when the amounts of membranes with VSVG-GFP are high, this might form some kind of a continuous parallel ribbon that is suitable for the VSVG-GFP diffusion.
4. The KAR model assumes that the pH gradient across the compartments can contribute to some of the asymmetry in the intra-compartment transport that is needed for the KAR model to work. However, it is not clear if the gradient is actually sufficiently sharp to produce the observed behavior, or if the cargo in general would behave as needed for the KAR model to work. A similar argument can be made with respect to the concept of the Ca^2+^ gradient. The presence of a pH gradient along the GA is well known, starting from 6.9 at the *cis* side and reaching 4–5 at the *trans* side, and this gradient is important for the sorting of secretory proteins [75]. Similarly, lipid gradients across the GA are described [51].

One ionic gradient *per se* (*i.e.*, the pH gradient) might be insufficient for the ionic-gradient-dependent multimerization of proteins. However, if the possibility of several ionic gradients is taken into consideration, this mechanism can be considered feasible. At least it is known that the Ca^2+^ in the ER is higher than in the GA, and that the Ca^2+^ in the GA is higher than that in post-Golgi compartments. The main problem would be whether pumps making ionic gradients are distributed, forming a gradient, or whether there is a single place where these are concentrated. It seems that the latter case is unsuitable because protons diffuse faster than proteins. However, additional analysis is necessary for clarification of this issue. Until now, it has not been clear what the real concentrations of K^+^, Na^+^ and Cl^−^ are in the ER and in the different compartments of the GA. The combination of several gradients here would potentiate this mechanism of sorting [76–78].

Finally, knowing that 30 min after the release of the 40 °C temperature block the kinetics of VSVG-GFP exit from the Golgi area show exponential decay [35] can be explained on the basis of the hypothesis that during the 30 min after the release of the 40 °C temperature block, several transport waves can pass through the GA, making the intercisternal connections wider and more clustered. Again, as above, it might be that when the amounts of VSVG are high, the membranes that contain it will form some kind of parallel cisternal network or additional “ribbon” (parallel to that formed by the enzymatic domains) through which the VSVG molecules can diffuse freely, whereas when the amounts of VSVG are small, the VSVG-positive structure does not form such a continuous ribbon.

Let us now consider Table 1. Here we assume that we have several consecutive compartments along the secretory pathway; namely, the ER, the intermediate compartment (IC), the *cis*-Golgi (*cis*), the medial Golgi (med1, med2, med3, med4), the TGN, and the post-Golgi compartment (PGC). Consecutive pairs of these compartments can be connected in two different ways: (1) ER–IC, *cis*–med1, med2–med3, med4–TGN; or (2) ER, ER exit sites–*cis*, med1–med2, med3–med4, TGN–PGC. Other combinations are not allowed. When these pairs are connected, across each of these pairs, a small ionic gradient is formed due to the presence of the continuous ionic gradient along the *cis*-*trans* direction. The concentration of an ion in any distal compartment in any of these pairs induces a significant change in the temporal oligomerization of a cargo protein. Temporal oligomerization means that the monomeric and oligomeric phases of our cargo proteins are in dynamic equilibrium, and that simultaneously both monomers and oligomers of different sizes can exist. If a tubule connecting two compartments is relatively thin, the monomer form of our protein can easily pass through this tubule. In contrast, the oligomeric forms can move, but much more slowly, depending on their size.

Assume also that the oligomeric forms of our protein are formed with a higher probability in solutions with higher concentrations of an ion. Under these conditions, the monomer concentration, which is higher in the proximal compartment, will move towards the distal compartment and form more oligomers there. The oligomers will diffuse more slowly to the proximal compartment, and as a result the concentration of our protein in the distal compartments becomes higher. For the sake of simplicity, let us assume that this effect in each pair is equal to 1 to 2. Now let us see how it works. In the ER, the concentration of our cargo is 10 conditional units, and our protein is constantly synthesizing. The upper row of Table 1 shows situation 1. Due to the above-mentioned reasons, the concentration of our cargo in the IC would be 2-fold higher than in the ER. The other compartments do not contain cargo, and are not important at this moment. Row 2 shows situation 2. The IC and *cis* are connected, so the 20 units, which were accumulated in the IC will be divided into 7 in the IC and 13 in *cis*. The third row demonstrates situation 1 again, where the IC accumulates 20 units of the protein from the ER. The 13 units that were accumulated in *cis* are now divided into 4 (*cis*) and 9 (med1), and so on. After 26 rounds of pumping, the concentration of our protein in the PGC reaches 50 units. At this moment, it can depart. And so on.

For Table 2, the assumptions are the same, with the only difference that after some (in our case—med4) compartment, the orientation of the vector of concentration of a Golgi enzyme is changed, being directed retrogradely.

## 6. Conclusions

Thus, today, the KAR model and the carrier maturation-progression model are the most promising for intra-Golgi transport. The latter can be considered as the asymmetrical KAR mechanism. Our analysis does not mean that the other models have been disproven by the existing evidence. We understand that the real question is not whether any model is exactly right because none of them are, but whether any is a mostly accurate qualitative description of the process. The KAR model explains not only all of the previous experimental evidence, which presents difficulties for the other models, but also the concentrations of albumin, the regulatory secretory proteins, and the GRPs in defined Golgi compartments, and furthermore, the rare presence of intercisternal connections.

## Figures and Tables

**Table 1:** Modeling the concentrations of soluble cargo across the Golgi apparatus according to the kiss-and-run model. ER: endoplasmic reticulum; IC: intermediate compartment; *cis: cis*-Golgi; med1, med2, med3, med4: medial Golgi; TGN: *trans*-Golgi network; PGC: post-Golgi compartment.

Number	ER	IC	*cis*	med1	med2	med3	med4	TGN	PGC
1	10	20	0	0	0	0	0	0	0
2	10	7	13	0	0	0	0	0	0
3	10	20	4	9	0	0	0	0	0
4	10	8	16	3	6	0	0	0	0
5	10	20	6	13	2	4	0	0	0
6	10	9	17	5	10	1	3	0	0
7	10	20	7	15	4	7	1	2	0
8	10	9	18	6	13	3	5	1	1
9	10	20	8	16	5	11	2	4	1
10	10	9	19	7	14	4	9	2	3
11	10	20	9	17	6	12	4	7	3
12	10	10	19	8	15	5	11	3	7
13	10	20	9	18	7	13	5	9	7
14	10	10	19	8	17	6	12	5	11
15	10	20	9	18	8	15	6	11	11
16	10	10	19	9	17	7	14	7	15
17	10	20	9	19	8	16	7	14	15
18	10	10	19	9	18	8	15	10	19
19	10	20	10	19	9	17	8	17	19
20	10	10	20	9	19	8	17	12	24
21	10	20	10	19	9	18	10	19	24
22	10	10	20	9	19	9	19	14	29
23	10	20	10	19	9	19	11	22	29
24	10	10	20	9	19	10	20	17	34
25	10	20	10	19	10	19	12	25	34
26	10	10	20	10	19	11	20	20	39
27	10	20	10	20	10	20	13	37	39
28	10	10	20	10	20	11	22	25	51
									Departure
29	10	20	10	20	10	21	16	31	25
30	10	10	20	10	20	12	35	19	37
31	10	20	10	20	11	21	15	29	37
32	10	10	20	10	21	12	24	22	44
33	10	20	10	20	11	22	15	31	44
34	10	10	20	10	21	12	35	25	49
35	10	20	10	20	11	22	20	40	49
36	10	10	20	10	21	14	28	29	60
									Departure
37	10	20	10	20	12	23	16	31	29
38	10	10	20	11	21	13	26	20	40
39	10	20	10	31	11	23	15	31	40
40	10	10	20	14	28	16	32	24	47
41	10	20	11	23	15	29	25	51	47

42	10	10	21	13	25	18	36	33	65
									Departure
43	10	20	11	23	14	29	23	46	33
44	10	10	21	12	25	17	35	26	53
									Departure
45	10	20	11	22	14	28	20	41	26
46	10	10	21	12	24	16	32	22	45
47	10	20	11	22	13	27	18	36	45
48	10	10	21	12	23	15	30	27	54
									Departure

The same color over two paired figures indicate the Golgi compartments, which are connected in the defined time. For instance, in row 1, Figures 10 and 20 in columns of ER and IC are pictured in blue. This indicates that ER and IC are connected, and due to this connectivity, albumin undergoes 2-fold concentration in IC. In the second row, Figures 7 and 13 in IC and *cis* compartments (columns) are colored in blue, indicating that IC and *cis* are connected and 20 molecules of albumin, which were in IC in the end of the first round of concentration (*cis* was empty), are redistributed into 7 and 13, giving almost 2-fold concentration in *cis* compartment. In the third row, figures 10 and 20 in the columns of the ER and IC are pictured in yellow, whereas figures 4 and 9 in the columns *cis* and med1 are colored in blue. This indicates that the ER and IC compartments are connected. The *cis* and med1 compartments are also connected. In both cases, due to connectivity, albumin undergoes 2-fold concentration. At the end of the second round of concentration, the *cis* compartment contained 13 molecules of albumin. The med1 compartment contained zero molecules. Due to connectivity 13 and the ion-dependent mechanism of concentration, molecules of albumin underwent redistribution into 4 and 9. A similar process is shown in the remainder of the table. In the 26th row, one can see the word “departure”. This reflects our assumption that when a significant level of cargo concentration was reached, the post-Golgi compartment could exit from the Golgi area. We assume that departure occurs if the concentration of our cargo in the PGC is higher than 5-fold. However, the departure does not affect the ability of the system to concentrate cargo and in six rounds of concentration, the matured (with 5-fold enrichment of cargo) post-Golgi compartment is ready again. Importantly, after the restoration of intra-Golgi transport, the number of concentration rounds, which are necessary for cargo concentration in PGCs until it reaches 5-fold, decreased and then reached the plateau.

**Table 2:** Modeling the concentrations of the Golgi glycosylation enzymes across the Golgi apparatus according to the kiss-and-run model.

ER	*cis*	med1	med2	med3	med4	med5	TGN	PGC
**1**	**2**	**1**	**2**	**1**	**2**	**2**	**1**	**1**
	**1**	**2**	**1**	**2**	**2**	**1**	**2**	**1**
10	10	10	10	10	10	10	10	10
10	20	7	13	7	13	13	7	10
10	7	18	7	13	17	9	11	6
10	20	8	17	10	20	13	7	6
10	9	19	9	18	22	11	9	4
10	20	9	19	13	27	13	7	4
10	10	19	11	21	27	13	7	4
10	20	10	20	16	32	13	7	4
10	10	20	12	24	30	15	7	4
10	20	11	21	18	36	15	7	4
10	10	21	13	26	34	17	7	4
**10**	**20**	**11**	**23**	**20**	**40**	**16**	**8**	**4**
10	10	21	14	29	38	18	8	4
9	18	12	23	22	45	17	9	4
9	10	20	15	30	41	21	9	4
8	16	12	23	24	49	20	10	4
8	9	19	16	31	46	23	9	5
7	14	12	23	26	51	21	11	5
7	8	16	16	33	48	24	11	5
6	12	14	28	27	54	23	12	5
6	9	17	15	30	52	26	11	6
5	10	11	21	27	55	25	12	6
5	7	14	16	32	53	26	12	6
4	8	10	20	28	57	25	13	6

In the first and the second rows the possible combinations of pairs connected with each other are shown. The consecutive compartments are aligned and can form two combinations of pairs, namely, (1) ER–*cis*-most cisternae, first medial cisterna–second medial cisterna, third medial cisterna–fourth medial cisternae, fifth medial cisterna–TGN; PGC is non-connected (the first row in Table 2); (2) ER is non-connected, *cis*–med1, med2–med3, med4–med5, TGN–PGC (the second row in Table 2). Until the fourth medial cisterna, the capacity of the next compartment to pump ions is thus higher than the capacity of the previous one. However, in the last two consecutive pairs next considered, namely third medial cisterna–fourth medial cisterna and fourth medial cisterna–*trans*-most cisterna, and in the more distal parts, the capacity of the distal compartments to pump protons is lower than the corresponding proximal ones. As in Table 1, the same colors indicate the Golgi compartments, which are connected in the defined time. We do not present here the situation when the Golgi compartments do not contain Golgi glycosylation enzymes, because without Golgi glycosylation enzymes the Golgi apparatus does not exist. Therefore, let us assume that in the beginning (when ionic pumps do not work), concentration of a Golgi glycosylation enzyme is equal to 10 in each compartment. During the first round of the concentration (the fifth row), when the first combination of compartment pairs is used, concentration of the enzyme increased in *cis*, med2, med4, med5 compartments (corresponding compartment pairs are colored in yellow, blue, green and yellow again). During the next round of the concentration (the sixth row of Table 2), when the second combination of pairs is used and the ER is isolated, concentration is increased in the following compartments: med1, med3, med4 (magenta, due to the inverted directionality of concentration), TGN. The third round of concentration (connected pairs are colored in yellow, green, blue, yellow) gives a concentration of the enzyme in the *cis* (yellow), med2 (yellow), med4 (blue), med5 (yellow). In such a way, concentration of the enzyme in the med4 compartment increases during every round of concentration reaching 5.7-fold after 23 rounds of concentration.

## Abbreviations

CPM model: compartment progression/maturation model
COP: coatomer protein
ER: endoplasmic reticulum
GA: Golgi apparatus
GRPs: Golgi resident proteins
Man: mannosidase
VSVG: G protein of the vesicular stomatitis virus
